# Surgical Trauma in Mice Modifies the Content of Circulating Extracellular Vesicles

**DOI:** 10.3389/fimmu.2021.824696

**Published:** 2022-01-18

**Authors:** Souren Mkrtchian, Anette Ebberyd, Rosanne E. Veerman, María Méndez-Lago, Susanne Gabrielsson, Lars I. Eriksson, Marta Gómez-Galán

**Affiliations:** ^1^ Department of Physiology and Pharmacology, Section for Anesthesiology and Intensive Care Medicine, Karolinska Institutet, Stockholm, Sweden; ^2^ Department of Clinical Immunology and Transfusion Medicine and Division of Immunology and Allergy, Department of Medicine, Solna, Karolinska University Hospital and Karolinska Institutet, Stockholm, Sweden; ^3^ Genomics Core Facility, Institute of Molecular Biology gGmbH (IMB), Mainz, Germany; ^4^ Function Perioperative Medicine and Intensive Care, Karolinska University Hospital, Stockholm, Sweden

**Keywords:** circulating extracellular vesicles, surgery, proteomics, miRNA, alpha-synuclein

## Abstract

Surgical interventions rapidly trigger a cascade of molecular, cellular, and neural signaling responses that ultimately reach remote organs, including the brain. Using a mouse model of orthopedic surgery, we have previously demonstrated hippocampal metabolic, structural, and functional changes associated with cognitive impairment. However, the nature of the underlying signals responsible for such periphery-to-brain communication remains hitherto elusive. Here we present the first exploratory study that tests the hypothesis of extracellular vesicles (EVs) as potential mediators carrying information from the injured tissue to the distal organs including the brain. The primary goal was to investigate whether the cargo of circulating EVs after surgery can undergo quantitative changes that could potentially trigger phenotypic modifications in the target tissues. EVs were isolated from the serum of the mice subjected to a tibia surgery after 6, 24, and 72 h, and the proteome and miRNAome were investigated using mass spectrometry and RNA-seq approaches. We found substantial differential expression of proteins and miRNAs starting at 6 h post-surgery and peaking at 24 h. Interestingly, one of the up-regulated proteins at 24 h was α-synuclein, a pathogenic hallmark of certain neurodegenerative syndromes. Analysis of miRNA target mRNA and corresponding biological pathways indicate the potential of post-surgery EVs to modify the extracellular matrix of the recipient cells and regulate metabolic processes including fatty acid metabolism. We conclude that surgery alters the cargo of circulating EVs in the blood, and our results suggest EVs as potential systemic signal carriers mediating remote effects of surgery on the brain.

## Introduction

There is a growing body of evidence that aseptic tissue injuries including surgery are closely associated with rapid onset of structural and functional changes within the brain, predominately in regions involved in cognitive processes, that are dependent on periphery-to-brain signaling pathways (1–4). Using an animal model of orthopedic surgery, we have previously shown a profound astrocytic response in the hippocampal area entailing morphological, metabolic, and functional alterations in neuronal circuits involved in cognitive processing, including synaptic transmission and plasticity (5, 6). However, the nature of the systemic signals traveling to the brain and triggering the aforementioned functional alterations remains largely unknown. We have suggested that the surgery-mediated activation of the innate immune system, which, within hours, orchestrates an adaptive systemic inflammatory response (7) might be responsible for such periphery-to-brain signaling. While such instantaneous activation of the immune system is suggested to produce rapid changes on the molecular and cellular levels in the brain, the periphery-to-brain communication pathways associated with more long-lasting cognitive and behavioral effects remain unknown.

Emerging evidence suggests an important role of extracellular vesicles (EVs) in inter-cellular-tissue communications including periphery-to-brain signaling (8–10). It may therefore be hypothesized that EV-dependent signaling provides an alternative pathway that is responsible for long-term changes in higher brain functions after surgery (5, 6).

Circulating EVs can be produced by a variety of tissues, however, one of the main sources is the blood immune cells. These EVs are suggested to serve as important modulators of innate and adaptive immune responses (11). In addition, several studies indicate that peripheral inflammation can modify the cargo of the immune cell-produced EVs that can in an endocrine-like manner regulate intracellular processes in the distant tissues, including the brain (12).

Here, in this first exploratory study, we have mapped the proteome and miRNAome of circulating EVs at different time points after mouse orthopedic surgery to establish a potential correlation/association with the temporal kinetics of the surgery-triggered systemic inflammatory response, brain dysfunction as previously described in a surgical animal model.

## Materials and Methods

### Animals

14–16 weeks old male C57BL6 (Janvier, Germany) mice were housed five per cage under temperature- and humidity-controlled conditions in a 12 h light/dark cycle and fed standard rodent chow and water *ad libitum*.

All experiments were approved by the Local ethics Committee for Animal Research of Stockholm North and Karolinska Institutet in Sweden.

### Surgery

The open stabilized tibia fracture was performed as previously described (5). Briefly, under the isoflurane anesthesia (2.1% inspired concentration in 0.30 FiO2) and analgesia (buprenorphine, 0.1 mg/kg, s.c.) a longitudinal incision was made on the left hind paw and the muscles were disassociated. A 0.38 mm stainless steel pin was then inserted in the intramedullary canal with the subsequent osteotomy. The wound was irrigated, sutured with 6-0 Prolene, and mice were allowed to recover in a warm box before returning to the home cage. The temperature was monitored and maintained at 37°C with the aid of a warming pad and temperature-controlled lights (Harvard Apparatus).

Animals from the surgery pool were randomly divided into three groups depending on the time point at which the blood sampling was performed: 6- (S6h), 24- (S24h), and 72 h (S72h) post-surgery. Each experimental group included 6 animals (in the mass spectrometry and NTA analysis the C6h group consisted of 7 animals). The S24h and S72h groups received a daily dose of analgesia to avoid possible effects of pain on the brain. Control mice received an equal volume of saline (s.c.). To avoid experimental variation over time, control animals from the same batch of animals were assigned to each of the groups (C6h and C24-72h) and received a daily injection of saline (s.c).

### Serum Sampling

Animals were deeply anesthetized with pentobarbital (0.1 ml, ip) and blood was collected from the left ventricle of the heart with a 23-G needle attached to a 1 ml syringe and immediately transferred to a 1.5-ml Eppendorf tube. Blood was allowed to coagulate at room temperature for 45 min and posteriorly centrifuged at 3000 g for 10 minutes at 4°C. Supernatants (sera) were stored at -80°C until further use.

### Isolation of Extracellular Vesicles

EVs were isolated using the commercial kit ExoQuick ULTRA (System Biosciences Inc., Mountain View, CA).

Following the manufacturer’s protocol serum samples were cleared by centrifugation for 10 min at 12 000 g and transferred to a new tube. Immediately after that 67 µl of the ExoQuick buffer A was added to the 250 µl of the cleared serum, the mix was incubated for 30 min at 4°C and centrifuged at 3000 g for 10 min at 4°C. The supernatant was saved for further analyses as the EV-depleted serum (dEVs). EV-containing pellet was resuspended in the 200 µl of Buffer B and EVs were isolated using the purification columns from the kit in 500 µl of elution buffer. Both the EV isolated fraction and the dEV serum were stored at ‐80°C until further use.

We have submitted all relevant data of our experiments to the EV-TRACK knowledgebase (EV-TRACK ID: EV210383) (13).

### Nanoparticle Tracking Analysis

The size and particle concentration of the serum EVs were determined by nanoparticle tracking analysis(NTA) using an LM10 platform with an sCMOS camera from NanoSight Ltd. The samples were diluted in sterile-filtered PBS to a particle concentration of 5x10^8^ – 5x10^9^ particles/ml and analyzed with camera level 14 and detection threshold 3. For each sample, four consecutive videos were recorded in RT while injecting the sample with a syringe pump (speed 50).

The average from the four consecutive videos for each sample was calculated and posteriorly used for the visualization of the results as Concentration (particles/ml) vs. Particle Size (nm).

Due to a large number of samples (N=31), the NTA analysis was performed in two different sessions (run 1 and run 2). In run 1 we included the S6h (N=6) group together with its corresponding control group (C6h, N=7). In run 2 we included C24-72h (N=6), S24h (N=6), and S72h (N=6).

### Negative Stain Transmission Electron Microscopy

Three microliters of the EV sample were applied on glow discharged carbon-coated and formvar stabilized 400 mesh copper grids (Ted Pella) and incubated for approximately 30s. Excess of the sample was blotted off and the grid was washed with MilliQ water prior to the negative staining using 2% uranyl acetate. Transmission electron microscopy (TEM) imaging was done using Hitachi HT7700 (Hitachi High-technologies) transmission electron microscope operated at 100 kV equipped with a 2kx2k Veleta CCD camera (Olympus Soft Imaging System).

### Small RNA Sequencing

RNA isolation. RNA was isolated from all groups (N=6 in each group). Total RNA was extracted from the 200 µl of EVs fractions using the Exosomal RNA isolation kit from Norgen Biotek Corp. (Nordic BioSite AB, Täby, Sweden). RNA was eluted in a 25 µl of elution buffer and RNA concentrations were determined by Qubit 4 fluorometer (Invitrogen).

Library preparation. RNA quality was controlled with Agilent Bioanalyzer 2100 (Agilent, Palo Alto, CA) equipped with the small RNA chip. Small RNA libraries were constructed using the NEXTFLEX^®^ Small RNA-Seq Kit v3 (Bioo Scientic Corp., Austin, Texas, USA) according to the manufacturer’s protocol. Libraries were prepared with a starting amount of 2.3 ng of RNA and amplified in 22 PCR cycles. Amplified libraries were purified by running an 8% TBE gel and size-selected for insert sizes of 15-40 nt (library sizes of 143-168 bp).

Sequencing. All samples were pooled in equimolar ratio and sequenced on the Illumina NextSeq 500/550 high output flowcell, with a 75- cycle kit, single read for 84 cycles plus 7 cycles for the index read. Library construction, quality control, and sequencing were completed at the Genomics Core Facility, Institute of Molecular Biology GmbH (IMB), Mainz, Germany.

Bioinformatic analysis. The raw sequence reads in FastQ format were cleaned from adapter sequences and size-selected for 14-34 base-long inserts (plus 8 random adapter bases) using cutadaptv.2.4 (http://cutadapt.readthedocs.org) with parameters ‘-j 8 -a TGGAATTCTCGGGTGCCAAGG -m 22 -M 42’ followed by quality checks with FastQC (https://www.bioinformatics.babraham.ac.uk/projects/fastqc). Read alignment to the mouse GRCm38/mm10 genome from Gencode release M25 (ftp://ftp.ebi.ac.uk/pub/databases/gencode/Gencode_mouse/release_M25) with concomitant trimming of the 8 random bases was performed using Bowtiev.1.2.2 (https://bowtie-bio.sourceforge.net).

For miRNA-focused analysis, the genome-aligned reads in SAM format were selected in the size range 20-24 bases using GNUAwkand Samtoolsv.1.10 (https://www.htslib.org), converted into sorted BAM files with Samtools, and read counts were summarized either per miRNA locus or per mature miRNA using featureCountsusing miRNA annotation either from Gencode M25 or from miRbase v.22 (ftp://mirbase.org/pub/mirbase/22/genomes/mmu.gff3). The miRbase annotation was converted from GFF3 into GTF format for use with featureCountsusing, the Bioconductor package rtracklayerin R v.3.6.0.

The expression of miRNAs was normalized as counts per million reads (CPM). The value of 10 CPM was chosen as a cut-off margin for filtering out the low-expression genes. Differential expression analysis was carried out with DESeq2 v.1.26.0 (https://bioconductor.org/packages/release/bioc/html/DESeq2.html) as implemented in the online tool iDEP (http://bioinformatics.sdstate.edu/idep) using a significance cut-off of 5% false discovery rate (FDR). Since no statistical difference was found between the two control groups (C6h and C24-72h), in order to simplify the miRNA differential expression analysis, only one set of controls (C6h, N=6) was selected and hereafter referred to as Control.

The fold change (FC) threshold for selecting differentially expressed genes was ≥1.5. Heat maps of differentially expressed genes and principal component analysis (PCA) using log2 normalized CPM expression values were generated using Qlucore Omics Explorer 3.2 (Qlucore, Lund, Sweden). Log2-transformed CPM values were used to generate Volcano plots (RStudio v 1.4.1717, PBC).

The miRNA sequencing data have been deposited in NCBI’s Gene Expression Omnibus (14) and are accessible through GEO Series accession number GSE115440).

### Liquid Chromatography-Tandem Mass Spectrometry–Based Proteome Analysis

Protein identification and quantification were carried out at the Proteomics Biomedicum core facility, Karolinska Institutet (https://ki.se/en/mbb/proteomics-biomedicum). Details of the sample preparation, peptide labeling with TMTpro mass tag reagent and subsequent separation of labeled peptides on EASY-Spray C18 column and mass spectra acquisition on Orbitrap Q Exactive HF mass spectrometer (ThermoFisher Scientific) are described in the **Supplementary File S1**. As the TMT-labeled experiments allow a maximum of 16 samples that could be analyzed simultaneously, the data were acquired from two independent runs, with C6h and S6h samples assigned for the first run, and the C24-72h, S24h and S72h samples assigned for the second run. Acquired raw data files were analyzed using Proteome Discoverer v2.4 (ThermoFisher Scientific) with Mascot Server v2.5.1 (Matrix Science Ltd., UK) search engine against mouse protein database (SwissProt). Initial search results were filtered with 5% FDR using the Percolator node in Proteome Discoverer. Quantification was based on the reporter ion intensities, which were log2 and quantile normalized and analyzed for the differential expression of proteins with GraphPad Prism (v. 9.1.1) using multiple unpaired t-tests analysis with FDR set to 5%. Quantile normalized and log2-transformed protein abundances were used to generate Volcano plots (RStudio v 1.4.1717, PBC).

The mass spectrometry proteomics data have been deposited to the ProteomeXchange Consortium *via* the PRIDE (15) partner repository with the dataset identifier PXD030167

### miRNA qPCR

TaqMan^®^ Advanced miRNA Assays (Applied Biosystems, ThermoFisher Scientific) were used for validation of miRNA-seq results. cDNA templates from the selected EV total RNA samples were prepared using the TaqMan^®^ Advanced miRNA cDNA Synthesis kit. The resulting cDNAs were amplified using 7500 Real-Time PCR System (Applied Biosystems, ThermoFisher Scientific) and the following TaqMan^®^ Advanced miRNA Assays: mmu-miR-143-3p (assay ID, mmu480935_mir), mmu-miR-499-5p (mmu482780_mir), mmu-miR-375-3p (mmu48114_mir), mmu-miR-1a-3p (mmu482914_mir), mmu-miR-541-5p (mmu481211_mir).

All samples were amplified in triplicate. Mmu-miR-103-3p was chosen as the housekeeping miRNA based on the analysis of expression stability of several miRNAs using the RefFinder tool (https://www.heartcure.com.au/reffinder/). The relative abundance of each miRNA was estimated according to the 2^–ΔΔCt^ method.

### Bead-Based Purification of EVs

Streptavidin-coated magnetic beads (SVMS-40-10, Spherotech) were coated with the biotinylated CD63 (clone MEM-259, BioSite Flow), CD9 (clone HI9a, Biolegend), and CD81 (clone M38, BioSite Flow) antibodies as it is described elsewhere (16). EVs (15 µg of total protein) from C24-72h and S24h groups were incubated overnight with antibody-coated beads. A sample with no EVs was included as a negative control. Beads were recovered on a magnetic stand, resuspended in RIPA buffer (Abcam) including protease and phosphatase inhibitors (Roche), sonicated, vortexed, and centrifugated at 10000 g. The supernatant containing EVs’ proteins was collected for the α-synuclein immunodetection.

### Western Blot

EVs, dEVs, and serum samples were solubilized with 2% SDS or RIPA buffer and thoroughly vortexed. Protein concentration was determined using the micro BCA Protein Assay Kit (Thermo Scientific) and samples containing equal amounts of protein (5 µg) were resolved by SDS-PAGE and transferred to a PVDF membrane (Invitrogen). Depending on the secondary antibodies (HRP- or IRDye-conjugated) protein bands were detected using either enhanced chemiluminescence reagents (GE Healthcare) and ChemiDoc MO analyzer (Bio-Rad) or Odyssey infrared fluorescence detection system (LI-COR, Lincoln, NE, USA).

The following primary antibodies were used at a 1:500 dilution: CD81 (sc-166029, Santa Cruz Biotechnology), HSP70 (ab181606, Abcam), CD63 (ab59479, Abcam), α-syn (610786, BD Bioscience).

### Statistics

For the statistical analyses of miRNA qPCR experiments we have used ANOVA one-way test followed by Dunnett’s multiple comparisons test (Graph Pad Prism v. v. 9.1.1). Data are presented as means ± SEM. P<0.05 was considered significant.

## Results

### Characterization of EVs

**Figure 1:**
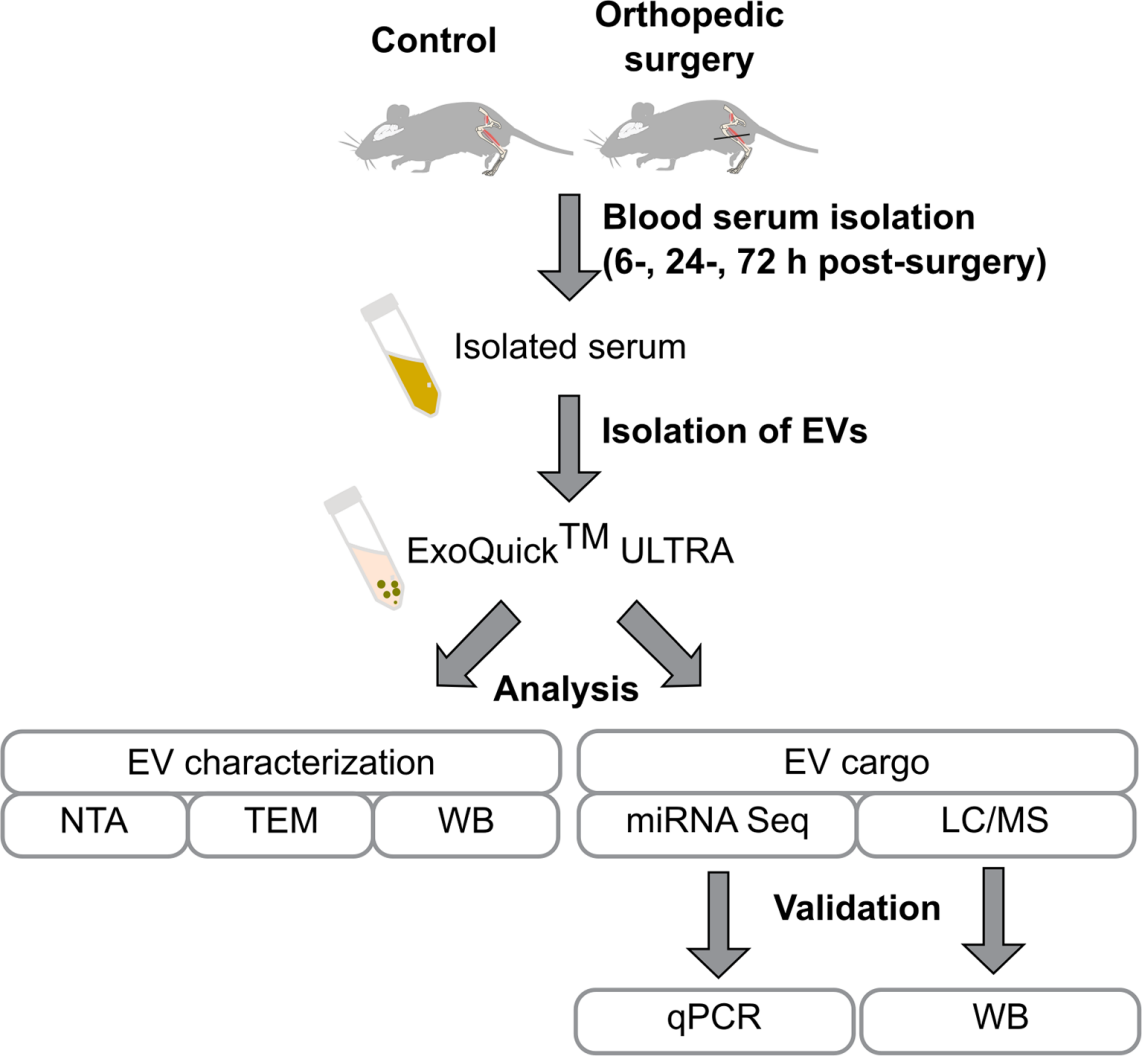
Flowchart of the study design. Mice were subjected to tibia surgery, blood was collected from the control and surgery animals after 6, 24, and 72 h post-surgery. Isolated blood serum was used for the purification of EVs using the ExoQuick ULTRA kit. EVs were characterized by Nanoparticle Tracking Analysis (NTA), transmission electron microscopy (TEM), and identification of EV enriched proteins by western blot. EVs’ proteome and miRNAome were investigated by LC/MS and RNA-seq and findings were validated by qPCR and western blot.

EVs were isolated from the blood serum with further characterization of EVs and analyses of the EV cargo as schematically presented in **Figure 1**.

Isolated EVs were characterized using different approaches: transmission electron microscopy (TEM), Nanoparticle Tracking Analysis (NTA), and immunochemical detection of EV enriched proteins (western blot).

**Figure 2:**
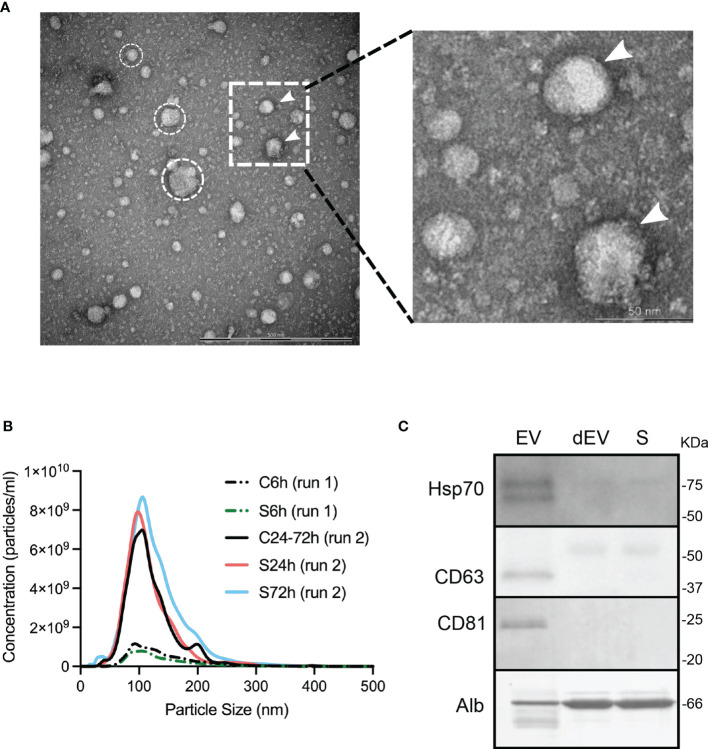
Characterization of circulating EVs. **(A)** TEM image of the isolated particles, scale bar, 500 nm. Encircled are representative spherical particles. White arrows indicate putative lipid bilayers of EVs. The dotted insert is represented in higher magnification on the right panel. **(B)** Representative size distribution profile of isolated particles and their concentrations estimated by NTA. However, no differences in the control vs. S6h and the control vs, S24h and S72h comparisons were detected. **(C)** Western blot identification of EV enriched proteins and albumin. SDS-PAGE for CD63 western blot was run under non-reducing conditions. dEV, serum depleted of EVs, S, serum.

TEM was performed in C24-72h (N=4) and S24h (N=4) groups and no differences were observed regarding the number, morphology, and size of the detected particles (data not shown). **Figure 2A** shows a representative TEM image from a control mouse demonstrating the presence of particles ranging from 30 to 150 nm, which is a common pattern for the EVs isolated using the ExoQuick kit (17). In addition, these particles appear to be encircled by the membranous structures, identifying them as typical extracellular vesicles (**Figure 2A**, right panel).

NTA analysis, which determines the size distribution and the number of isolated particles (particles/ml), was performed on all samples but in two different sessions (run 1 or run 2; see Methods). The size distribution of the isolated EVs’ particles was similar for all the experimental groups, independently of the assigned session. In all cases, the averaged particle size distribution ranged between 50-200 nm with the size of the most frequently detected particle (mode) close to 100 nm (**Figure 2B**). The average concentration of the particles was also similar between the control and surgical groups within the same session (run1: C6h vs S6h and run 2: C24-72h vs S24h and S72h). However, we found considerable differences in the number of particles detected during the different sessions (**Figure 2B**). This difference is probably due to the intrinsic high variability associated with the NTA method itself, which makes this method not suitable for the comparison of particle concentrations between the groups.

Finally, two EV enriched proteins, CD63 and CD81, known to be located on the surface of EVs, and an intra-vesicular protein, HSP70 were identified in the lysed EVs by western blot (**Figure 2C**). At the same time, the abundant serum protein, albumin was substantially depleted from the EV fraction (**Figure 2C**). It can be concluded that all the above-mentioned EV characteristics including the size and morphology of isolated particles, and the presence of EV enriched proteins are identical to the ones described by other groups using similar isolation methods (17).

### Surgery Effects on the Proteome of Serum EVs

The EV proteome was investigated using the LC-Tandem Mass Spectrometry approach (for details see *Materials and Methods* and **Supplementary File S1**). Analysis of the acquired data identified 343 proteins in the samples from the first run (C6h and S6h) and 417 proteins in the samples from the second run (C24-72h, S24h and S72h) (see *Materials and Methods*) (**Supplementary Tables S1**, **S2**). Although several of them can be related to the pool of common serum proteins (albumin, apolipoproteins, and various chains of immunoglobulins), the gene ontology (GO) analysis identified many GO cellular component terms that can be specifically associated with EVs (**Table 1**). The most prominent EV-related groups are the late endosome lumen, blood microparticle, and the integrin alpha9-beta1 complex. The latter includes three integrins: Itgβ1, Itgβ3, and Itgα2b, characteristic EV surface proteins that facilitate the interaction of EVs with the extracellular matrix of the target tissues and even function as organotropic cues (18).

**Table 1:** Gene ontology (cellular component category) analysis of the differentially expressed EV proteins at 24 h post-surgery.

Gene onthology_cell component	Fold enrichment	FDR
late endosome lumen (GO:0031906)	54.02	3.83E-02
other organism part (GO:0044217)	54.02	3.79E-02
membrane attack complex (GO:0005579)	54.02	1.02E-07
fibrinogen complex (GO:0005577)	54.02	1.34E-06
myosin II filament (GO:0097513)	54.02	3.04E-03
integrin alpha9-beta1 complex (GO:0034679)	54.02	3.75E-02
blood microparticle (GO:0072562)	43.22	2.55E-08
spherical high-density lipoprotein particle (GO:0034366)	40.52	4.12E-06
intermediate-density lipoprotein particle (GO:0034363)	36.02	6.07E-04
proteasome core complex, alpha-subunit complex (GO:0019773)	33.77	8.03E-05
extrinsic component of external side of plasma membrane (GO:0031232)	30.01	1.19E-04

Proteins are ranked according to the fold enrichment of the corresponding cellular component term (http://geneontology.org/).

**Figure 3:**
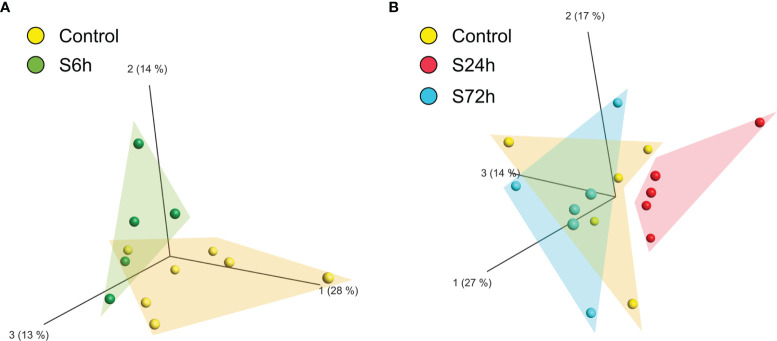
Principal component analysis (PCA) of the proteomic data of circulating EVs. LC/MS produced quantitative proteomic data (protein abundances) were quantile normalized, log2-transformed, and used by Qlucore software for creating the PCA plots. **(A)** Control and 6 h post-surgery (S6h) experimental group form individual clusters. **(B)** Control and S24h groups are completely separated whereas the S72h group overlaps with the control samples.

**Figure 4:**
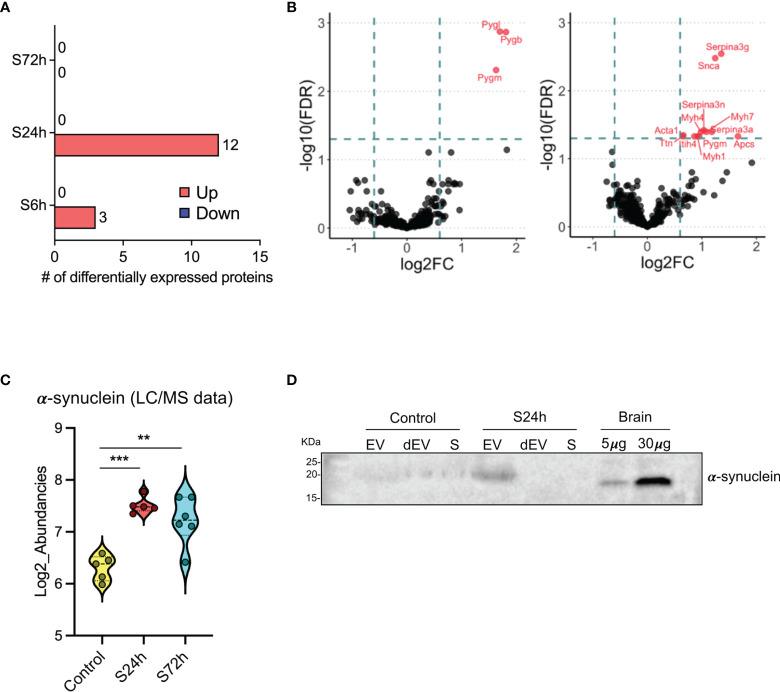
Differential expression of proteins in the post-surgery circulating EVs. **(A)** Bar diagram representing the number of differentially expressed proteins in the 6, 24, and 72 h post-surgery groups compared to the control values as assessed by GraphPad Prism (v. 9.1.1) using multiple unpaired t-tests analysis with FDR < 0.05 and fold change (FC) > 1.5. (*Materials and Methods*). **(B)** Volcano plots showing the differentially expressed proteins at 6h (left) and 24h (right) post-surgery compared to their corresponding control groups (C6h and CS24-72h). Dotted lines represent FC > 1.5 threshold (log2-FC) and FDR <0.05 (-log10FDR), respectively. **(C)** Differential expression of α-synuclein in the EVs from S24h and S72h groups using log2-transformed LC/MS data. **(D)** α-synuclein expression validation by western blot. Mouse brain (hippocampus) homogenate was used as a positive control. SDS-PAGE was run under non-reducing conditions. dEV, serum depleted of EVs, S, serum. Snca in the 24h Volcano plot is the official gene symbol for α-synuclein (α-syn). **p < 0.005, ***p < 0.0005.

Comparison of the different experimental groups using the principal component analysis (PCA) demonstrates separation of the 6- and 24 h post-surgery group from the corresponding control samples, C6h and C24h-72h, respectively (**Figure 3**). Based on these data it was expected that most of the differentially expressed proteins would appear in the 24 h samples. Indeed, the expression levels of 12 proteins were found to be significantly different from the controls (FC>1.5, FDR<0.05) (**Figure 4A**, **Supplementary Table S3**). In addition, three different isoforms of glycogen phosphorylase (PYG) were also differentially expressed at 6 h post-surgery, of which the muscle isoform remains up-regulated even after 24 h (**Figure 4A** and **Supplementary Table S3**). These data can be better represented by the Volcano plots that enable quick visual identification of the up-and down-regulated proteins (**Figure 4B**). Interestingly, one of the top up-regulated proteins in the 24 h group was α-synuclein (α-syn), a protein whose expression is largely associated with neural tissues (right panel in **Figures 4B, C**).

To confirm the EV origin of the α-syn we have analyzed serum, EVs, and dEVs samples from C24-72h and S24h groups by western blot. The α-syn signal was detected in the EV fraction from the 24 h surgical mouse, whereas it was absent in the serum and dEVs samples (**Figure 4D**). In the EVs from the control mouse, the signal was weak but detectable in all the samples. These data are complemented by yet another western blot analysis of the EVs that were additionally purified using magnetic beads coated with CD63, CD9, CD81 antibodies (see *Materials and Methods*). α-syn was identified in such enriched EVs from both control and 24 h surgical groups (**Supplementary Figure S1**). However, contrary to the MS data, we did not observe similar quantitative changes between the control and surgical mice. This is probably due to several reasons, such as the semiquantitative nature of the western blot and the difficulty to accurately quantify the low protein amounts after the EV enrichment.

### Surgery Effects on the miRNAome of Serum EVs

**Figure 5:**
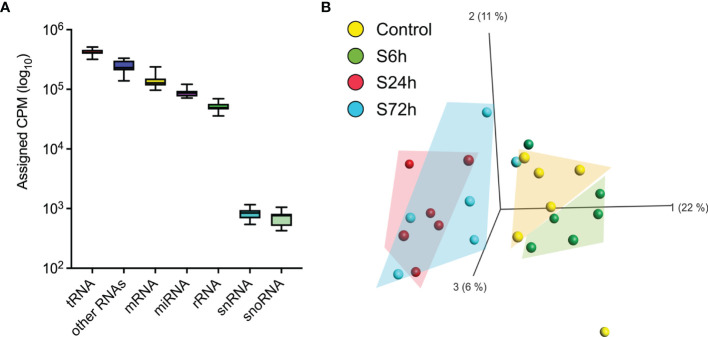
RNA mapping and miRNA PCA. **(A)** The averaged RNA-seq data from the control samples were used to map RNA categories [sRNAbench webserver (19)]. **(B)** Principal component analysis of the circulating EVs’ miRNA data. Normalized miRNA read counts [Counts Per Million reads (CPM)] were log2-transformed and used by Qlucore software to create a PCA plot. 24 h and 72 h post-surgery groups cluster together and are separated from the control whereas the 6 h group clusters with the control.

**Figure 6:**
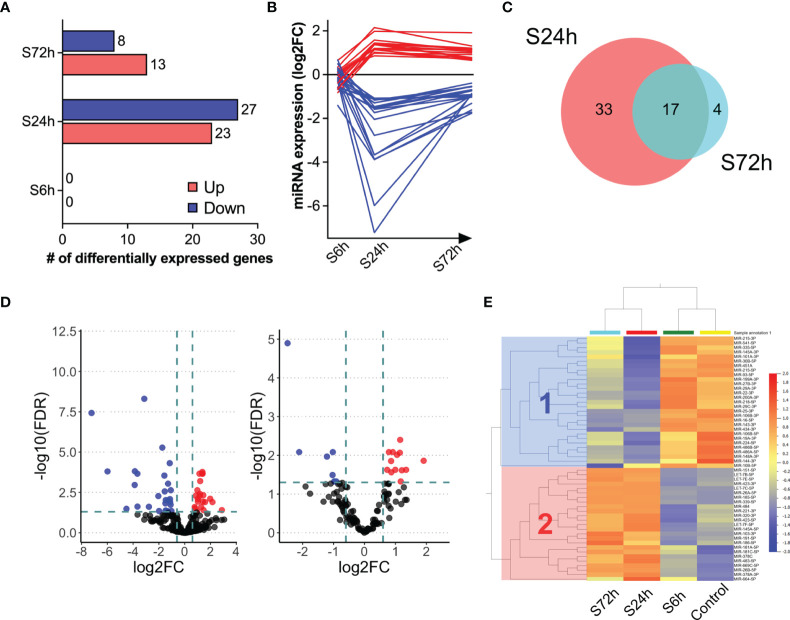
Differential expression of miRNAs in the post-surgery circulating EVs. **(A)** Bar diagram representing the number of differentially expressed miRNAs in the 6, 24, and 72 h post-surgery groups compared to control values as assessed by DESeq2 analysis (FDR<0.05 and fold change (FC)>1.5; see *Materials and Methods*). **(B)** Post-surgery temporal kinetics of the differentially expressed miRNAs. Each line represents an individual differentially expressed miRNA. **(C)** Volcano plots showing the differentially expressed proteins at 24- (left) and 72 h (right) post-surgery compared to the control group (Control). The dotted lines represent FC > 1.5 threshold (log2-FC) and FDR <0.05 (-10logFDR), respectively. **(D)** Venn diagram showing the overlap between the differentially expressed miRNAs at 24 and 72 h post-surgery. **(E)** The log2-transformed CPM values of differentially expressed miRNAs at 24 and 72 h and the same miRNAs from the control and S6h groups were used to generate heatmap (Qlucore). Cluster 1 (blue) and cluster 2 (red) include down-regulated and up-regulated miRNAs at 24 and 72 h, respectively.

Next-generation sequencing of small RNA libraries detected a mix of various RNA species including approximately 10% of mature miRNAs (**Figure 5A**). After removing miRNAs below the expression threshold (see *Materials and Methods*) the remaining 252 miRNAs were chosen for further analysis (**Supplementary Table S4**). PCA demonstrates clear separation of the control samples from the S24h and S72h groups, whereas the S6h group was clustered closer with the control samples (**Figure 5B**). At the same time, there is a partial overlap between the 24 and 72 h groups. Further analysis confirmed this trend, revealing the presence of 50 dysregulated miRNAs (FC>1.5, FDR<0.05) in the EVs from the S24h group (**Supplementary Table S5**). The surgery effect declines after 72 h as judged by the lower number of dysregulated miRNAs (21) observed in the S72h group (**Figure 6A** and **Supplementary Table S5**). These data are graphically interpreted by the plot showing temporal kinetics of the fold changes of dysregulated miRNAs with peak values at 24 h, which decrease after 72 h almost to the baseline levels (**Figure 6B** and **Supplementary Table S5**), and by the corresponding Volcano plots (**Figure 6C**). Interestingly, only four out of 21 dysregulated miRNAs at 72 h post-surgery were not differentially expressed at 24 h (**Figure 6D**), which indicates the lasting, though declining effect of surgery on miRNA expression.

The heatmap presented in **Figure 6E** offers an even more nuanced view of the surgery effects. Thus, in addition to the already shown PCA clustering of control and experimental groups (control/C6h and S24h/S72h), the hierarchical clustering of miRNAs reveals two distinct clusters where cluster 1 includes down-regulated, and cluster 2, up-regulated at 24- and 72 h miRNAs.

**Figure 7:**
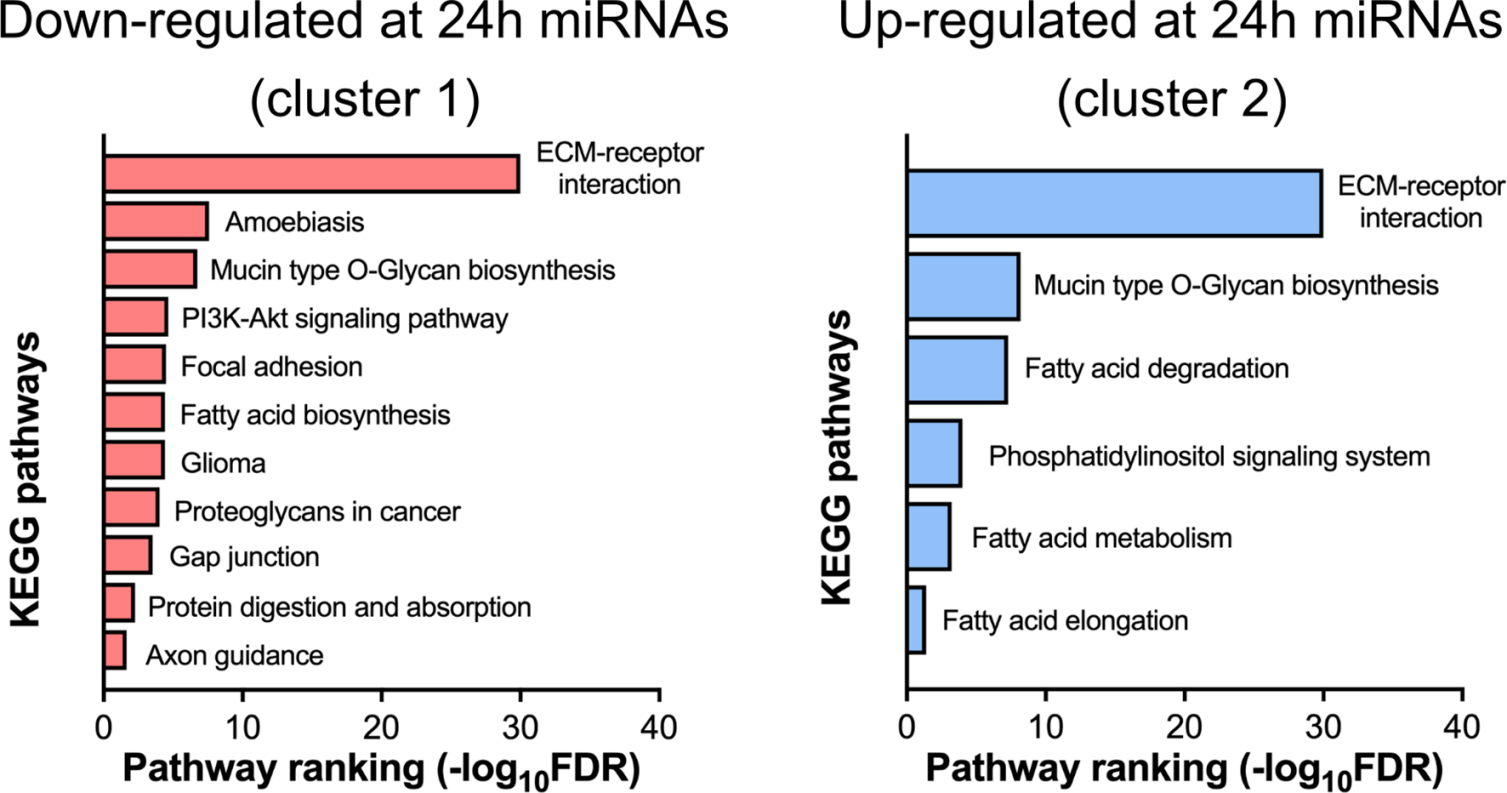
Pathway analysis of differentially expressed miRNAs. Down- (cluster 1 from **Figure 6E**) and up-regulated (cluster 2 from **Figure 6E**) at 24 h post-surgery miRNAs were analyzed by the miRPath online tool (see *Materials and Methods*) that determines the miRNA target mRNAs (based on experimental data) and analyses the overrepresentation of these genes in the different KEGG pathways.

miRNAs from circulating EVs are suggested to be involved in the post-transcriptional regulation in the target tissues (20). The miRNAs from both clusters were examined by the DIANA-miRPath online tool (21) that combines the prediction of miRNA target mRNAs (based on experimental data) with the analysis of the extent of enrichment of these genes in the different biological pathways. The bar graphs in **Figure 7A** show several KEGG pathways that are predicted to be regulated by the miRNAs from both clusters. A number of these pathways are involved in lipid metabolism, ECM-receptor interaction, gap junction, and various signal transduction pathways.

In order to validate the miRNA sequencing findings, we have estimated the expression of several differentially expressed miRNAs using the qPCR approach. Overall, the pattern of changes of the expression levels for the selected miRNAs was similar between the RNA-seq and qPCR analyses (**Supplementary Figure 2**).

**Figure 8:**
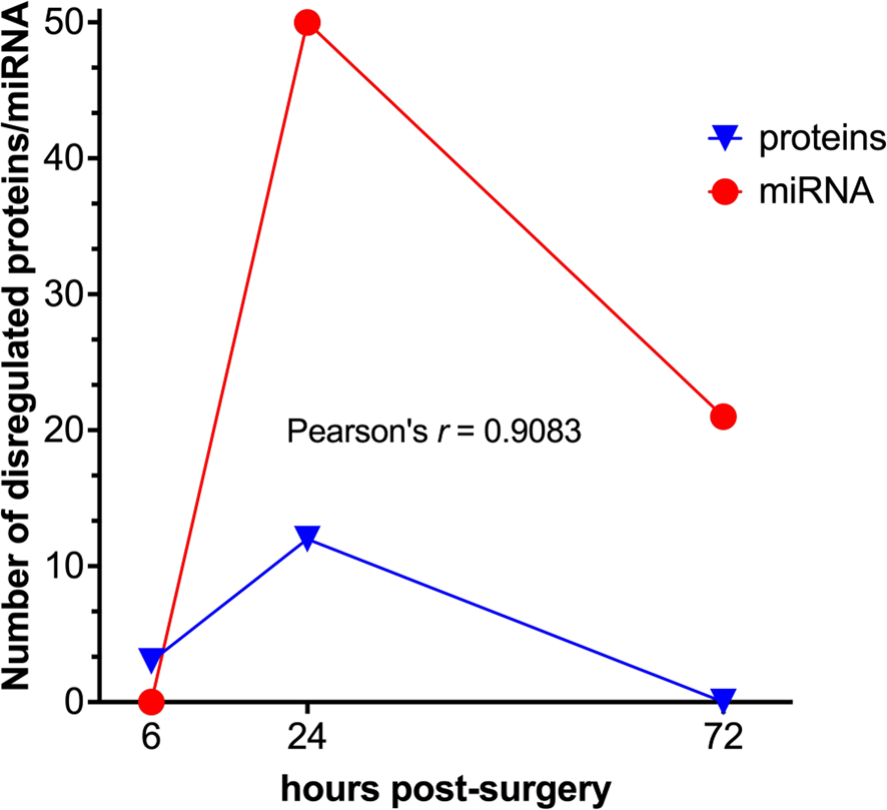
Correlation analysis between the differentially expressed protein and miRNA data sets. Temporal kinetics of the differential expression of EVs’ proteins and miRNAs demonstrates a high degree of correlation as to the Pearson’s linear correlation coefficient (GraphPad Prism).

Finally, it should be concluded that there is certainly a synergism in the temporal kinetics of the changes in the expression levels of both EV cargo molecules, proteins, and miRNA, as judged by the significant correlation between the numbers of both classes of dysregulated molecules at each post-surgery time point (**Figure 8**).

## Discussion

The principal aim of the present study was to determine whether surgical trauma, as represented by the mouse orthopedic surgery model is capable of modifying the cargo composition of circulating EVs, which would constitute a first step towards the elucidation of the potential role of EVs as active systemic mediators of surgery-induced periphery-to-brain communication.

We have, indeed, found statistically significant differences in the proteome and miRNAome of circulating EVs within the first 72 h after surgery. Their temporal appearance coincides with both the pattern of initiation and resolution of the systemic cellular and humoral inflammatory response and with the brain structural, metabolic, functional, and behavioral changes observed after orthopedic surgery in mice (5–7).

Proteomic profiling of the circulating EVs revealed that three proteins, i.e., brain, skeletal muscle, and liver isoforms of PYG, were rapidly up-regulated after surgery (**Figure 4B**). PYG is an enzyme involved in the breakdown of glycogen to glucose and as such essential for the maintenance of glucose homeostasis. The expression of PYG isozymes is not strictly tissue-specific (22), therefore it is difficult to pinpoint the precise tissue origin of EVs in our study.

Nevertheless, the surgery-injured muscles can be suggested as the most probable source of EVs carrying these proteins. Similarly, the increased levels of the same set of proteins were detected in the circulating EVs following yet another tissue injury (cardiac muscle) after the chemotherapy treatment (23). Therefore, the release of these EV-localized PYG isoforms appears to be an early indicator of tissue injury. In addition, the enrichment of EVs by PYGs might also occur due to the increased glycogenolysis in the muscle and liver, which is often observed during stress (24).

Alternatively, the increase of the brain isoform of PYG in the EVs may reflect the metabolic state of the brain after surgery. Indirect evidence confirming this hypothesis is our data on the modified glucose and glutamate metabolism in the hippocampus accompanied by changes in lactate levels at the same time points after surgery (6).

The most prominent changes in the EVs’ protein cargo occur at 24 h post-surgery when 12 proteins were found up-regulated. Mining the functions and tissue expression profiles of these proteins allows conditional division of this group into two clusters: proteins related to the muscle structure and function [Myosin Heavy Chain 1, 4, and 7 (Myh1, Myh4, Myh7), PYGm, Titin (Ttn), Actin, Alpha Skeletal Muscle (Acta1)] and proteins involved in the acute-phase response to stress [Serpina3g, Serpina3n, Serpina3a, Serum Amyloid P-Component (Apcs), Inter-Alpha-Trypsin Inhibitor Heavy Chain H4 (Itih4)]. The muscle-related cluster represents the same tendency that was detected already at the 6 h time point, that is the profound disruption of the surrounding tissues, including the muscle upon tibia surgery. Considering the known activation of the inflammatory response after surgery (25, 26) the elevation of levels of many acute-phase proteins from the second cluster is also not surprising. For instance, the circulating levels of Itih4 were found elevated after the surgical trauma as shown using yet another animal model of surgery (27). It can be therefore suggested that post-surgery EVs might also spread inflammatory signals to remote organs.

One of the differentially expressed proteins at 24 h post-surgery is α-syn, which cannot be categorized into either of these two subgroups. α-syn is expressed predominantly in neurons and mostly localizes at the presynaptic terminal where it regulates vesicle turnover (28). The aggregated form of α-syn is a hallmark of a group of neurodegenerative diseases, including PD and dementia with Lewy bodies (DLB) (29). However, α-syn is also expressed in peripheral tissues, including muscle cells (30) and blood cells, such as erythrocytes and platelets (31–33) whereas lesser amounts are found in peripheral blood mononuclear cells (PBMC) (34). This suggests potential source tissues of the α-syn found in the circulating EVs. One possibility is that it is released into EVs from the damaged muscles along with the abovementioned muscle proteins. It can also be hypothesized that PBMCs activated after surgery can shed “inflammatory” EVs carrying α-syn as one of the cargo molecules.

Up-regulation of α-syn in the circulating EVs after surgical trauma prompts an intriguing hypothesis that associates EV-born α-syn with acceleration and/or exacerbation of neurodegenerative diseases (35, 36) and the risk for late-onset dementia (37, 38) after surgery. Consistent with this hypothesis different preclinical and clinical studies demonstrated that α-syn bearing EVs are capable of amplifying and propagating PD-related pathology (39–42). In addition, it was also reported that LPS-induced peripheral inflammation may potentiate the delivery of erythrocyte-produced EVs containing α-syn across the BBB (43).

However, further detailed studies are certainly needed to explore this hypothesis and decipher possible clinical implications of α-syn up-regulation in the circulating EVs after surgery.

Whereas the protein fraction of EV cargo can potentially provide more information on the tissue origin of EVs, the miRNAs endowed with a strong post-transcriptional regulation potential are important for understanding and predicting the phenotypic changes of the recipient cells and tissues. It should be noted that despite the same temporal pattern of differential expression (**Figure 8**) the number of dysregulated miRNAs at 24 h post-surgery is significantly higher than the number of proteins at the same time point. Moreover, these changes do not completely subside as in the case with proteome but remain at a certain, albeit lower level at 72 h post-surgery, which might be indicative of a functionally more important role of EVs’ miRNAs as compared to proteins, with a wider temporal window of action.

Analysis of the miRNA differential expression at 24 h post-surgery revealed an explicit pattern of biological pathways that these miRNAs (via their target mRNAs) may affect in the putative recipient tissue(s). Thus, the set of down-regulated miRNAs is involved in several pathways, the majority of which are unified by a common theme - regulation of extracellular matrix (ECM) (**Figure 7**). Interestingly, this set includes only few miRNAs out of 27 down-regulated miRNAs (mmu-miR-29c-3p, mmu-miR-145a-3p, mmu-miR-148a-3p, mmu-miR-29a-3p, mmu-miR-25-3p) (**Supplementary Figure S3**, left panel). Nearly the same set of miRNAs is also associated with a much broader scope pathway, the PI3K/AKT/mTOR signaling. The ECM components targeted by miRNAs are represented by a group of various collagens and their cell receptors, namely integrins. Notably, different members of the human miR-29 family that were found down-regulated in this study, are known to have anti-fibrotic properties (44) due to their capacity to interact with many mRNAs coding for ECM proteins. It can therefore be suggested that down-regulation of these miRNAs in the EVs could stimulate the synthesis of ECM proteins in the target tissue(s), a process that is closely associated with wound healing, especially at the initial stages (45). In line with this, it was reported that various cargo molecules of circulating EVs including miRNAs are capable of modulating this pathway (46), and therefore might facilitate healing at the injury site.

Such up-regulation of ECM components is reminiscent of ECM re-modeling upon the metastatic process when tumor EVs are preparing the “soil” for the arriving cancer cells - a metastatic niche formation (47, 48). Considering the involvement of the same set of miRNAs in the regulation of PI3K/AKT/mTOR pathway (**Supplementary Figure S3**, left panel) it is tempting to speculate that such “soiling” of the recipient tissue might represent an important first step facilitating the following activation of the PI3K/AKT/mTOR system with the very same miRNAs. However, due to the immense complexity of this pathway and uncertain target tissues of post-surgery EVs, it is difficult so far to predict specific miRNA-mediated effects *via* such a mechanism.

In addition to the 27 down-regulated EV miRNAs, 23 up-regulated miRNAs were found associated with several KEGG pathways. Again, it is just a few miRNAs in this group that are driving most of the regulatory functions. These are four different mmu-*let* miRNAs and mmu-miR-339-5p, mmu-miR-423-5p that are involved in several pathways linked to the fatty acid metabolism, along with yet another group of miRNAs (mmu-miR-181a-5p, mmu-miR-26b-5p, mmu-miR-26a-5p, mmu-miR-378a-3p, mmu-miR-181c-5p, mmu-miR-378c) that is connected with the phosphatidylinositol signaling system (**Supplementary Figure S3**, right panel). The same group of *let* miRNAs can also regulate the ECM (**Supplementary Figure S2**, right panel). Interestingly, the same pathway is targeted by down-regulated miRNAs (see above), however, *let* miRNAs can modulate translation of only a few collagen mRNAs, whereas the down-regulated miRNAs target many more ECM components including integrin receptors.

As indicated above, most pathways linked to the up-regulated miRNAs are related to the fatty acid metabolism and involve mRNAs coding for both the enzymes participating in the fatty acid catabolism and the elongation and saturation of fatty acids. Therefore, it can be predicted that the overall effect of the miRNA up-regulation in the EVs would be the suppression of the fatty acid catabolism in the putative recipient tissue(s).

Interestingly, consistent with this prediction, unpublished hippocampal gene expression and metabolomic data from our laboratory indicate partial replacement of glucose as an energy source by fatty acids at 6 h post-surgery, whereas these changes were largely reversed 72 h after surgery and lipid catabolism was even decreased.

As in the case of down-regulated miRNAs, the group of up-regulated miRNAs is involved in the modulation of yet another complex multifunctional signal transduction pathway, phosphatidylinositol-dependent signaling. Similar to the PI3K/AKT/mTOR pathway, this signaling cascade is an active actor in many physiological and pathological intracellular events, which, on one hand, allows prediction of the active functional role of the post-surgery EVs in the various distant tissues, but, on the other hand, makes it equally difficult to envision any specific downstream effects.

In both up- and down-regulated miRNA groups, the miRPath pathway analysis tool singled out only a few dysregulated miRNAs that were considered to drive main metabolic effects. However, it cannot be ruled out that other differentially expressed miRNA might also modify gene expression in target cells. For instance, one of the top (FDR ranking) down-regulated at 24 h mmu-miR-143-3p is predicted to be involved in lipid and carbohydrate metabolism. Yet another top down-regulated mmu-mir-541-5p target is predicted to modify the MAPK signaling pathway (results not shown).

It should be mentioned that this study is not devoid of certain limitations. Thus, it is difficult to separate the effects of surgery and anesthesia without a corresponding experimental group of animals subjected only to anesthesia. We have previously demonstrated that the systemic immune activation in response to orthopedic surgery in mice is independent of the isoflurane anesthesia (7, 49). Nevertheless, it is still an open question if the modification of EVs composition under the same conditions is following the same pattern.

Another confounding factor is the use of only male animals in the study. We have unpublished yet data where we could not detect any sex differences in the behavioral experiments using the same tibia surgery model.

Proteomic data indicated α-synuclein as differentially expressed in the 24 h surgery samples. Western blot validation revealed a protein band slightly larger in size as compared to the brain positive control (**Figure 4D**). We believe that this size difference can be attributed to the different tissue sources of the protein and/or to the potential posttranslational modification(s) (50). However, more sensitive methods, such as immunoelectron microscopy might be needed to confirm the presence of the protein in the EVs.

Finally, there is an ongoing debate concerning the choice of the EV isolation method. The choice is largely dictated by the tissue type, available amounts, throughput options, suitability for various downstream applications, and the research question to be addressed. Taking all of these and also many comparative studies (17, 50–54) into account, we chose to use the commercial kit ExoQuick ULTRA. The ULTRA version enriches for EVs and has a relatively high yield, important for the small sample volumes obtained from mice. The disadvantage of using the ExoQuick, as well as similar precipitation methods is that it might produce certain amounts of contaminating proteins and also miRNAs associated with lipoproteins. However, whereas the gold standard EV isolation method, the ultracentrifugation could have provided with more refined EV fraction, it would had been not possible to apply using the minute amounts of mouse blood serum. The same reasoning can be applied to the other methods, such as size-exclusion chromatography (SEC), density gradient, affinity chromatography, *etc.* Interestingly, a comparison of SEC with the ExoQuick ULTRA using the identical serum volumes (250 µl) demonstrated the isolation of nearly the same set of EV-related proteins (17).

With the current study, we began to explore the hypothesis of circulatory EVs as potential mediators of the physiological effects of surgical trauma. The main question of the current project, whether the surgery is capable of modifying the expression of cargo molecules in circulating EVs, can be answered now positively. Moreover, differential expression of a specific set of miRNAs in the “surgical” EVs suggests that they can be involved in the regulation of several metabolic pathways, such as ECM-receptor interaction and lipid metabolism in the recipient tissues, which is consistent with our data on hippocampal metabolic and functional dysregulation after surgery (5, 6). Another interesting finding is the increased levels of α-syn in the circulating EVs after surgery, which might have potential long-term neurological consequences. We conclude that surgery alters the EV composition in the blood, which paves the way for further studies on the functional effects of these EVs on the brain.

## Data Availability Statement

The datasets presented in this study can be found in online repositories. The names of the repository/repositories and accession number(s) can be found below: NCBI GEO, accession no: GSE189972; PRIDE ProteomeXchange, accession no: PXD030167.

## Ethics Statement

All experiments were reviewed and approved by the Local ethics Committee for Animal Research of Stockholm North and Karolinska Institutet in Sweden.

## Author Contributions

Conception and design: MG-G, SM. Methodology support: MM-L, RV. Acquisition data: MG-G, AE. Analysis and interpretation of the data: MG-G, SM. Funding acquisition: LE. Writing outline of the manuscript: MG-G, SM. Reading and revision of the manuscript: all authors. All authors contributed to the article and approved the submitted version.

## Funding

This work was funded by The Swedish Medical Research Council and The Swedish Brain Foundation.

## Conflict of Interest

Author SG has a patent on B cell derived exosomes in immune therapy and is part of the Scientific Advisory Board of Anjarium Biosciences. RV is currently employed by Strike Pharma.

The remaining authors declare that the research was conducted in the absence of any commercial or financial relationships that could be construed as a potential conflict of interest.

## Publisher’s Note

All claims expressed in this article are solely those of the authors and do not necessarily represent those of their affiliated organizations, or those of the publisher, the editors and the reviewers. Any product that may be evaluated in this article, or claim that may be made by its manufacturer, is not guaranteed or endorsed by the publisher.
