# Supplementary material for: Surgical Trauma in Mice Modifies the Content of Circulating Extracellular Vesicles
Source: Front Immunol. 2022 Jan 18;12:824696. doi: 10.3389/fimmu.2021.824696 (PMC8804340; doi:10.3389/fimmu.2021.824696)
Supplement: Supplementary file 1 [file DataSheet_1.docx]

**Liquid Chromatography-Tandem Mass Spectrometry method description**

*Sample preparation*

Isolated EVs were dried in the vacuum concentrator (Eppendorf) and resolubilized in 12.5 µl of 20% SDS and 37.5 µl of 50 mM triethylammonium bicarbonate (TEAB), pH 8.0 to get a final concentration of 5% SDS. Following sonication in water bath for 5 min, proteins were reduced with 2 µl of 0.5 M dithiothreitol, incubated at 37°C for 30 min and then alkylated with 6 µl of 0.5 M iodoacetamide in dark for 30 min. Protein digestion was performed in S-Trap micro filters (Protifi, Huntington, NY) following the manufacturer’s recommendations. Briefly, samples were acidified with 7 µl of phosphoric acid to a final concentration of ca. 1% and diluted eight times with binding buffer (90% methanol and 100 mM TEAB, pH 7.1). Protein solutions were loaded onto a S-Trap micro filters by centrifugation at 4000 rpm for 30 s, and then the filters were washed four times with the binding buffer. Finally, 5 µl of 0.2 µg/µl of the trypsin solution in 50 mM TEAB, pH 7.1 was loaded onto the filters in a 1:20 proportion (enzyme:protein). Proteins were digested for 2 h at 47°C. To elute the peptides, three stepwise solutions were loaded, including 50 mM TEAB, pH 7.1, 0.2% formic acid (FA) and 50% acetonitrile (ACN)/ 0.2% FA. Eluted peptides were desalted on C-18 using HyperSep filter plate with 40 μL bed volume (ThermoFisher Scientific) and dried in vacuum concentrator prior to TMT labeling.

TMTpro mass tag reagent kit (ThermoFisher Scientific) was used for peptide labeling. Digested proteins (2 µg aliquot each) were resolubilized in 40 µl of 50 mM TEAB, pH 7.1 and 20 µg TMTpro reagents (dissolved in dry ACN) were added in a scrambled order and incubated at RT for 2 h. The reaction was stopped by addition of hydroxylamine to a concentration of 0.5% and incubation at RT for 15 min before samples were combined and cleaned on a C-18 HyperSep plate with 40 µl bed volume (ThermoFisher Scientific).

*Liquid Chromatography-Tandem Mass Spectrometry Data Acquisition*

The reconstituted peptides in solvent A (0.1% FA in 2% ACN) were separated on a 50 cm long EASY-Spray C18 column (ThermoFisher Scientific) connected to an Ultimate 3000 nano-HPLC (ThermoFisher Scientific) using a gradient from 2-26% of solvent B (98% AcN, 0.1% FA) in 90 min and up to 95% of solvent B in 5 min at a flow rate of 300 nl/min. Mass spectra were acquired by the Orbitrap Q Exactive HF mass spectrometer (ThermoFisher Scientific) in m/z 375 to 1500 at resolution of R=120,000 (at m/z 200) for full mass, followed by data-dependent higher energy collisional dissociation (HCD) fragmentations of 18 of the most intense precursor ions with a charge state 2+ to 7+. The tandem mass spectra were acquired with a resolution of R=60,000, targeting 2x105 ions, setting quadrupole isolation width to 1.4 Th and normalized collision energy to 33.

*Data Analysis*

Acquired raw data files were analyzed using Proteome Discoverer v2.4 (ThermoFisher Scientific) with Mascot Server v2.5.1 (Matrix Science Ltd., UK) search engine against mouse protein database (SwissProt). A maximum of two missed cleavage sites were allowed for full tryptic digestion, while setting the precursor and the fragment ion mass tolerance to 10 ppm and 0.02, respectively. Carbamidomethylation of cysteine was specified as a fixed modification. Oxidation on methionine, deamidation of asparagine and glutamine, as well as acetylation of N-termini and TMTpro were set as dynamic modifications. Initial search results were filtered with 5% FDR using Percolator node in Proteome Discoverer. Quantification was based on the reporter ion intensities.
