# Supplementary material for: Surgical Trauma in Mice Modifies the Content of Circulating Extracellular Vesicles
Source: Front Immunol. 2022 Jan 18;12:824696. doi: 10.3389/fimmu.2021.824696 (PMC8804340; doi:10.3389/fimmu.2021.824696)
Supplement: Supplementary file 2 [file DataSheet_2.pdf]

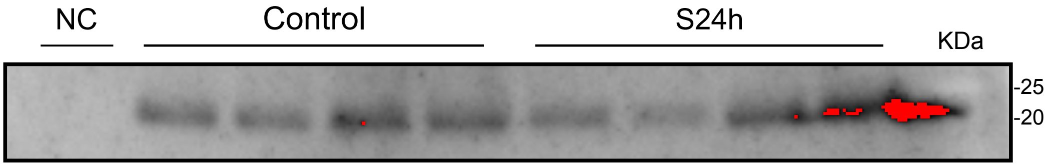

Supplementary figure 1. Validation of MS identification of the α-synuclein expression in the serum EVs. Serum EVs from the control and S24h groups were additionally purified by the bead-based method (see Methods and Materials) and subjected to the western blot using α-synuclein antibodies. SDS-PAGE was run under non-reducing conditions. NC, negative control.

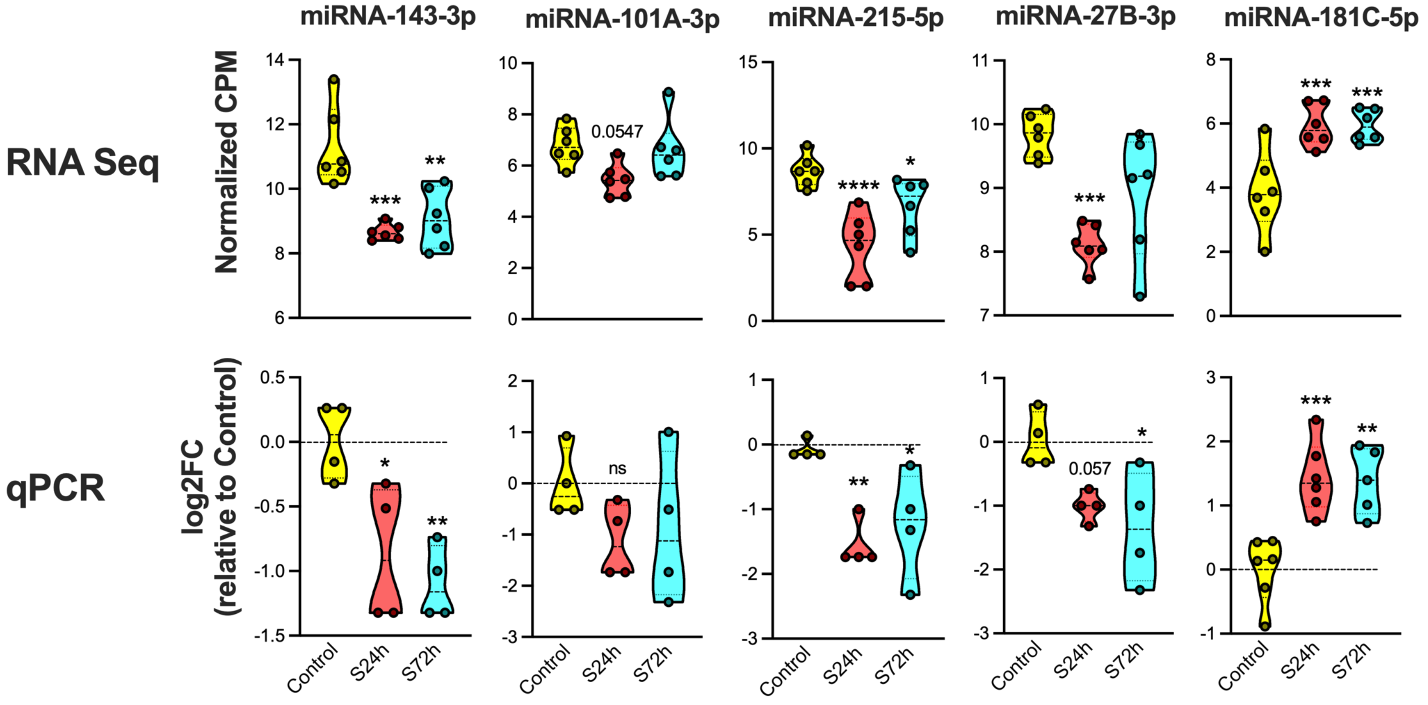

Supplementary figure 2. Validation of RNA-seq data. Expression of selected miRNAs was tested using the qPCR approach (see Materials and Methods). Upper panel, RNA-seq based miRNA expression data. Lower panel, expression of the same miRNAs as to the qPCR data.

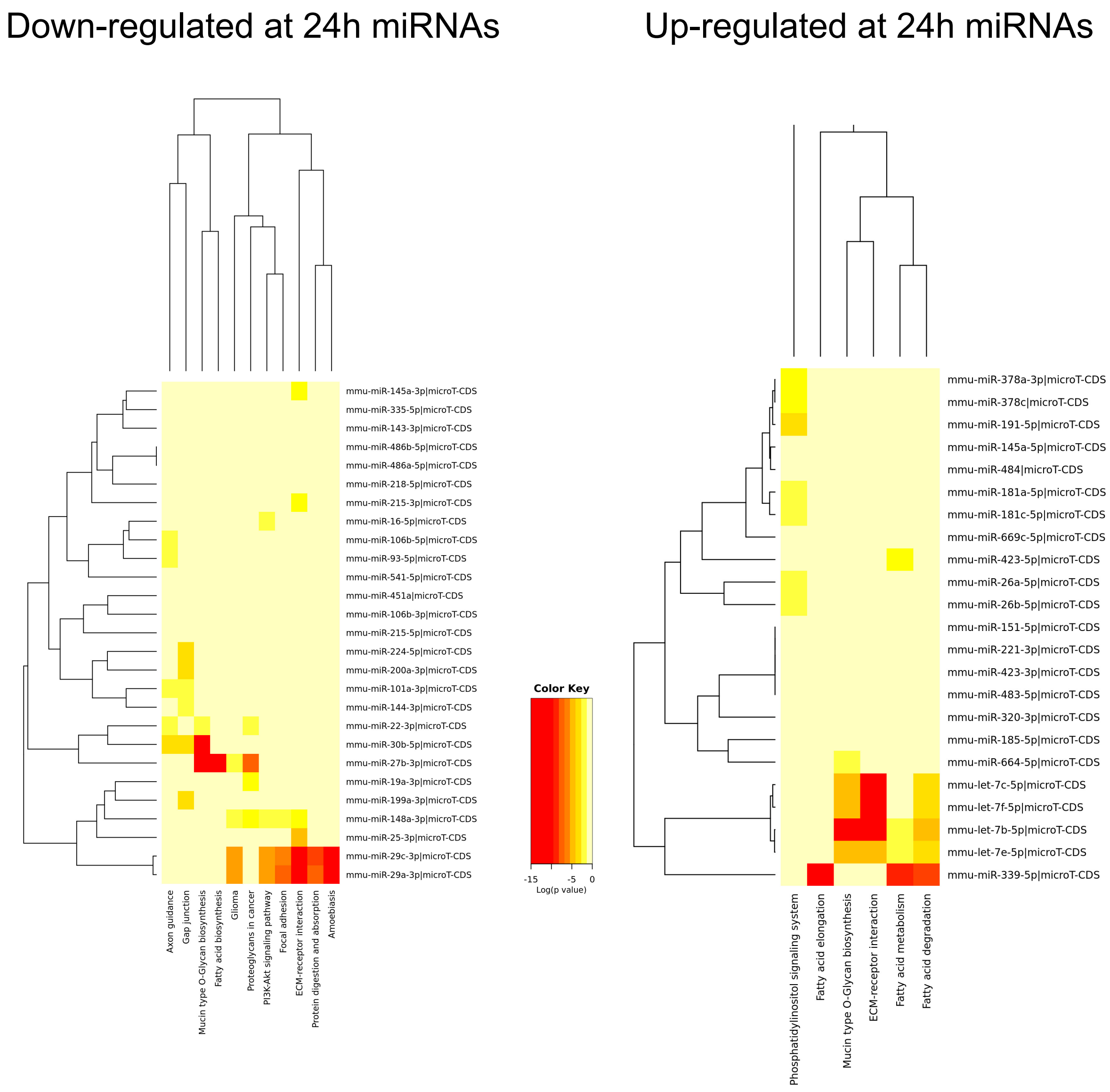

Supplementary figure 3. Association of differentially expressed miRNAs with KEGG pathways. Left panel, heatmap depicting the level of enrichment with KEGG pathways of the down-regulated at 24 h post-surgery miRNAs. Right panel, identical heatmap constructed for up-regulated miRNAs. In the center, color code bar. Heatmaps were produced using the Diana miRPath online tool (see Materials and Methods).
